# The *NARCONON*™ drug education curriculum for high school students: A non-randomized, controlled prevention trial

**DOI:** 10.1186/1747-597X-3-8

**Published:** 2008-03-19

**Authors:** Richard D Lennox, Marie A Cecchini

**Affiliations:** 1 Psychometrics Technologies, Incorporated, 2404 Western Park Lane, Hillsborough, NC 27278, USA; 2 Independent Research Consultant, 10841 Wescott Avenue, Sunland, CA 91040, USA

## Abstract

**Background:**

An estimated 13 million youths aged 12 to 17 become involved with alcohol, tobacco and other drugs annually. The number of 12- to 17-year olds abusing controlled prescription drugs increased an alarming 212 percent between 1992 and 2003. For many youths, substance abuse precedes academic and health problems including lower grades, higher truancy, drop out decisions, delayed or damaged physical, cognitive, and emotional development, or a variety of other costly consequences. For thirty years the Narconon program has worked with schools and community groups providing single educational modules aimed at supplementing existing classroom-based prevention activities. In 2004, Narconon International developed a multi-module, universal prevention curriculum for high school ages based on drug abuse etiology, program quality management data, prevention theory and best practices. We review the curriculum and its rationale and test its ability to change drug use behavior, perceptions of risk/benefits, and general knowledge.

**Methods:**

After informed parental consent, approximately 1000 Oklahoma and Hawai'i high school students completed a modified *Center for Substance Abuse Prevention (CSAP) Participant Outcome Measures for Discretionary Programs *survey at three testing points: baseline, one month later, and six month follow-up. Schools assigned to experimental conditions scheduled the Narconon curriculum between the baseline and one-month follow-up test; schools in control conditions received drug education after the six-month follow-up. Student responses were analyzed controlling for baseline differences using analysis of covariance.

**Results:**

At six month follow-up, youths who received the Narconon drug education curriculum showed reduced drug use compared with controls across all drug categories tested. The strongest effects were seen in all tobacco products and cigarette frequency followed by marijuana. There were also significant reductions measured for alcohol and amphetamines. The program also produced changes in knowledge, attitudes and perception of risk.

**Conclusion:**

The eight-module Narconon curriculum has thorough grounding in substance abuse etiology and prevention theory. Incorporating several historically successful prevention strategies this curriculum reduced drug use among youths.

## Background

### Effective education is needed to address today's burgeoning substance abuse problem

Although the annual, benchmark study, *Monitoring the Future *(MTF) [1], has measured small declines in drug use during the past few survey years, the estimated 13 million youths aged 12–17 in the U.S. who become involved with alcohol, tobacco and other drugs annually remains high compared with the declining trend seen during the 1980's which ended in 1992 [2].

Problem areas include the estimated $22.5 billion that underage consumers spent on alcohol in 1999 (of $116.2 billion total) [3]; an alarming 212 percent increase in the number of 12- to 17-year olds abusing controlled prescription drugs between 1992 and 2003; and youth initiation of pain relievers estimated at 1,124,000 in 2001, second only to marijuana initiation at 1,741,000 [2]. Controlled prescription drugs (including OxyContin, Valium and Ritalin) are now the fourth most abused substances in America behind only marijuana, alcohol and tobacco.

When prevention efforts fail it is not at small cost. In 2005, lifetime prevalence rates for any drug use were 21%, 38%, and 50% in grades 8, 10, and 12, respectively [1]. Although it can be argued that not all students who try drugs will develop problems, in 2002 the alcohol abuse and dependence-related costs for lost productivity, health care, criminal justice, and social welfare were estimated at $180.9 billion [4].

For many youths, substance abuse precedes academic problems such as lower grades, higher truancy, lower expectations, and drop out decisions [5]. In fact, the more a student uses cigarettes, alcohol, marijuana, cocaine and other drugs, the more likely they will perform poorly in school, drop out [6,7] or not continue on to higher education [8].

Consistent with the goals and public health agenda of the Office of National Drug Control Policy (ONDCP) and the Department of Education, the Narconon program's ultimate goal is to prevent and eliminate drug abuse in society. Research has shown that preventing or delaying initiation of alcohol or other drug use during early adolescence can reduce or prevent substance abuse and other risk behaviors later in adolescence and into adulthood [9,10]. However, there is still much discussion regarding what policy and strategies to employ toward this goal.

For the past 30-years, Narconon drug prevention specialists have delivered seminars aimed at supplementing existing prevention efforts by further illustrating materials covered in school curricula. In 2004, Narconon International developed an eight-module drug education curriculum for high school ages based on the research and writings of L. Ron Hubbard as incorporated into the secular Narconon drug rehabilitation methodologies. Program developers analyzed post-program student feedback, surveys collected as a quality management practice that has been in place since program inception and continues today, in light of evidence-based practices and prevention theory to create a stand-alone, universal (all youths) drug education curriculum for high school ages aimed at addressing key problem areas.

The eight module Narconon drug education curriculum for high school ages incorporates a unique combination of prevention strategies with content addressing tobacco, alcohol, marijuana and common "hard drugs." Health motivation, social skills, social influence recognition and knowledge-developing activities address a number of risk and protective factors in the etiology of substance abuse and addiction. The aim of this study was to assess the program's ability to change drug use behavior, attitudes and knowledge among youths and evaluate the components of the Narconon drug prevention curriculum against prevention theory.

## Methods

### Description of the sample

The Narconon program recruited 14 schools from two states. Schools were assigned to education or control groups based on similarity of school size, community size and general ethnicity. Schools also agreed to complete three testing points: Baseline, approximately one month later, and a six month follow-up. The full Narconon drug education curriculum was implemented either after completion of the baseline survey (education condition) or after completion of the final six month survey (control condition). Fidelity of curriculum delivery was verified by facilitator report.

After obtaining parental consent, there were 236 control group and 244 experimental group students in Oklahoma, with 295 control group and 220 experimental group students in Hawai'i. Voluntary assent and confidentiality were explained to the students. After the baseline survey, one charter school of 26 participants withdrew from the study for scheduling reasons. No provision was made to adjust representation by gender or potentially interesting ethnic or risk groups.

The study protocol and consent forms were reviewed and approved by Copernicus Group IRB (Protocol HI001). Human participant protections certified survey staff assigned each student a unique identification number based on a classroom roster. For confidentiality, students marked their answers on standard bubble answer forms labeled only with their unique identification number. The roster and identification code was used to give students the same identification number at each survey point, thus permitting comparison of answers given on each measurement occasion – a sampling strategy that provided the necessary statistical power to identify differences in tested variables among a universal classroom population, where the majority of youths do not use drugs. Completed answer forms were placed by each student into a security envelope, sealed, and returned to survey staff for mailing to the Principal Investigator for scanned data entry, data management, and statistical analysis.

### Drug education intervention

The study design called for each of the schools recruited to the experimental conditions to receive the complete drug education curriculum. Professionally trained facilitators followed a codified delivery manual and completed a daily compliance report. Codified Narconon drug prevention curriculum materials help the facilitator implement the program according to specific standards, maintaining program fidelity.

### Outcome measures

The primary outcome measure was last 30-day substance use using the *Center for Substance Abuse Prevention (CSAP) Participant Outcome Measures for Discretionary Programs *designed for outcomes evaluation in CSAP funded substance abuse prevention programs which is recommended for use in a pre-test/post-test design. (Form OMB No. 0930-0208 Expiration Date12/31/2005) [11]. Questions were directed to frequency of use of twenty two drugs of abuse including twelve questions from the Monitoring the Future Survey [1].

Secondary outcomes assessed by the CSAP instrument included perception of risk, attitudes and decisions about drug use including five questions from the Monitoring the Future Survey that ask about perceived harm from substance use; and four questions from the Student Survey of Risk and Protective Factors [11] that ask about drug use attitudes. In addition to calculating change in behavior and beliefs among individuals, these questions permit comparisons to state and national norms.

Additionally, the program developers recommended 25 questions that were appended to the CSAP survey for the purpose of assessing whether drug education concepts covered by the Narconon program are correctly understood by each program recipient, to what extent they are retained at follow-up points, and whether or not students could apply key program concepts. The program developer questions were designed to examine proximal effects including the ability of the program to educate by examining recall of program material, as well as give an impression of student capacity to apply program skills such as self-reported ability to communicate their beliefs on substance use, recognize and resist pressures to use substances, and make decisions.

### Statistical analysis

The non-randomized design – where it cannot be assumed that groups assigned to experimental and control conditions will be equal – calls for a conservative analysis. For this reason the study utilized Analysis of Covariance (ANCOVA) of the change scores from baseline, controlling for initial drug use as well as changes in the school populations as covariates. The autocorrelation among the classroom clusters was statistically accommodated through use of a nested treatment effect, in which the treatment effect was nested within the classroom effect. Type III sum of squares deviations between the baseline characteristics of both groups were used in all post-treatment statistical comparisons of the treatment and control group, thus statistically controlling for any differences existing at baseline and removing any effects caused by pre-existing differences between the two test conditions that might confound the results. In this way, the analysis is aimed at establishing the statistical strength and reliability of assigning any measured differences at the six-month post-treatment follow-up to the drug education received by the experimental group rather than any attempt to quantify those changes.

## Results

### Evaluation of Narconon curriculum components

Table 1 outlines the eight curriculum sessions against key constructs used by many drug prevention programs. The interactive curriculum imparts science-based information from fields as diverse as toxicology, forensic science, nutrition, marketing, pharmacology, and many others. Program materials include audiovisual support and clear lesson plans that are to be delivered in their entirety combined with quality management tools such as anonymous student questionnaires for each session and a facilitator's log sheet to list any session problems and/or questions. Facilitator training emphasizes the importance of effective communication as well as creating an environment in which students may ask questions, discuss personal situations, and actively participate.

**Table 1:** Constructs in the Narconon Drug Education Curriculum for high school students.

**Narconon Prevention Program Module:**	**Session 1: "Drugs and The Body"**	**Session 2: "What is a Drug?"**	**Session 3: Review** **Take Home Assignment: "Speaking With Your Parents."**	**Session 4: "Ecstasy – The Real Story"**	**Session 5: "Alcohol, Drugs and the Media"**	**Session 6: Review** **Take Home Assignment: "Examples of Drug Promotion"**	**Session 7: "Goals and the Emotional Scale"**	**Session 8: "Setting and Achieving Goals"**
**Knowledge objective**	Drugs have long-lasting physical consequences including deposition in tissues [34]; [35] with residual physical and psychological effects [36]; [37,38].	Medications, both licit and illicit, have a range of dose-dependent actions from stimulation to depression to death [39]. Drugs affect nutrient status [40]; deficiencies [41,42] can exacerbate withdrawal symptoms [43] and adversely affect mood [44].	Recap and review of previous modules and take home assignment results.	Media influence and marketing strategies aimed at youths impact their value systems and create false norms [45]. Contrast with negative effects of ecstasy [46], [47].	Further explores drug promotion strategies, particularly the prevalence of alcohol and tobacco advertising aimed at youth – often subtly placed [48].	The effects of drugs on the mind; a network of communications visual mental imagery and perceptions.	Impart the effects of drugs on a person's emotions [49] in contrast with satisfaction achieved from setting and achieving personal goals.	Recap and review of all previous modules.
**Social influence skills**	Resistance based on negative impact on health.	Resistance based on negative impact on health.	Recognize the influence of family and peers on drug use behaviors and establishment of norms.	Correction of false norms regarding popularity and positive attitudes toward substance use.	Recognize the influence of culture, media, technology and other factors on drug use behaviors.	Resistance based on negative impact on the mind.	Resistance based on negative impact on emotions.	Resistance based on negative impact on personal goals.
**Interactive ** **activity(ies)**	"Icebreaker" drill Question and answer session Take-home assignment: personal observations, parental discussion.	Orientation drill Question and answer session	Group discussion: findings from take home assignment. Group participation: Drugs and the nervous system. Group participation: effect of drugs on life.	Orientation drill Question and answer session	Orientation drill Question and answer session Take-home assignment: Find pro-drug advertising examples in in-store displays, magazines, movies, sporting or music events.	Group participation: Effects of drugs on the mind. Group discussion: findings from take home assignment. Group participation: "The media game."	Orientation drill Question and answer session	Orientation drill Class discussion: Setting doable goals Group participation: The scale of emotions. Essay: Goals for the coming year, how to get started.
**Competency enhancement**	Ability to use interpersonal communication skills.	Demonstrate the ability to assess valid information.	Ability to use interpersonal communication skills.		Cognitive skills for resisting media influences.	Ability to use interpersonal communication skills.	Demonstrate the ability to use decision-making skills and goal-setting skills.	Skills for increasing self-control and self-esteem realized through development of a clear set of personal goals and strategies for achieving those goals.
**Multi-component (family/community)**		Assignment involves parent-student conversation.	In 1998, six percent of parents reported never talked to their children about drugs, this doubled to 12 percent in 2004 [50].			Assignment involves recognition of messages seen in community settings.		

### Tests for selection bias: Demographic representation and drug use characteristics of groups at baseline

A total of 995 students out of a possible 1106 were recruited based on informed parental consent. Of these 726 completed both the baseline assessment and the six-month follow-up. The main sources of attrition were students not available on the day of survey and students no longer enrolled at the study school at the six month follow up.

Although selection of sites for "no treatment" attempted to match the demographic composition at intervention sites with respect to residence state, age, and general economic group, this strategy does not guarantee that the two types of sites are free from selection bias. Table 2 presents demographics composition of the control and treatment groups. Students frequently indicated several ethnic categories. The ethnic make-up of this group is particularly interesting as the evaluation includes a number of typically under-represented groups; however, the size and scope of this study do not make analysis of individual ethnic groups feasible.

**Table 2:** Demographics.

Subgroup	Count for Control Group	Count for Drug Ed Group
Male	319	171
Female	89	200
Black or African American	13	12
Asian	187	103
American Indian	39	51
Native Hawaii	60	113
Other Pacific Islander	36	37
White	220	215
Hispanic or Latino	21	32
Alaska Native	4	4
Other	18	26
Age 12 years old	0	0
Age 13 years old	0	0
Age 14 years old	17	62
Age 15 years old	98	119
Age 16 years old	148	122
Age 17 years old	125	51
Age 18 years old	24	25
Age 19 years old	3	0
Age 20 years old	1	0

The drug use portion of this questionnaire determines general *usage levels *for the various drugs (except for cigarettes and smokeless tobacco). For example, "On how many occasions during the last 30 days have you used marijuana ..." is answered on the scale: "1" = 0 occasions, "2" = 1–2 occasions, "3" = 3–5 occasions, "4" = 6–9 occasions, "5" = 10–19 occasions, "6" = 20–39 occasions, and "7" = 40 or more occasions. From this, Table 3 shows the means for both groups to be slightly higher than 1 or "0 occasions", indicating some degree of drug use but a high proportion of individuals not using substances, or that substance.

**Table 3:** Drug use at baseline: Comparison of means between treatment and control groups.

	Control Group *N *= 523	Drug Ed Group *N *= 435	Significance Level		
Drug Use Variable	Mean	Mean	*t*	*df*	*p *value^*a*^

B1 Cigarettes (frequency)	1.38	1.45	-0.962	850	0.336
B2 Smokeless tobacco	1.38	1.34	0.634	951	0.526
B3 Cigarettes (amt. smoked)	1.51	1.62	-1.047	956	0.295
B4 Alcohol	1.64	1.51	1.611	955	0.107
B5 Being drunk	1.28	1.31	-0.408	866	0.684
B6 Marijuana	1.24	1.22	0.326	944	0.744
B7 Marijuana (amt. smoked)	1.19	1.21	-0.332	894	0.740
B8 Sniffed glue	1.11	1.11	0.132	876	0.895
B9 LSD	1.03	1.05	-0.672	802	0.502
B10 Amphetamines	1.07	1.13	-1.404	731	0.161
B11 Crack	1.05	1.07	-0.836	804	0.403
B12 Cocaine	1.06	1.07	-0.247	922	0.805
B13 Tranquiller	1.08	1.10	-0.513	790	0.608
B14 Barbiturates	1.08	1.10	-0.699	732	0.485
B15 Crystal Meth	1.04	1.07	-0.829	769	0.407
B16 Amphetamine w/o Rx	1.09	1.08	0.022	907	0.982
B17 Heroin	1.02	1.05	-1.078	654	0.281
B18 Other Narcotics	1.07	1.08	-0.257	866	0.798
B19 Ecstasy	1.07	1.06	0.463	904	0.644
B20 Roofies	1.02	1.06	-1.087	588	0.277
B21 GHB	1.02	1.04	-1.126	532	0.261
B22 Super K	1.02	1.05	-1.013	626	0.311

^*a *^Controlling for baseline differences by using an analysis of covariance with a Type III sums of squares, The t-test df is corrected for unequal variances.

Comparison of the means on the drug use measures between the treatment and control groups prior to receiving any drug education, as seen in Table 3, show that the two groups do not differ significantly on any of the drug abuse measures, suggesting that any difference seen at follow-up was unlikely to be caused by pre-existing differences.

### Effects of the Narconon drug education curriculum on drug use compared with sites that have not yet received the curriculum

At follow-up, as shown in Table 4, students in the drug education program, but not the control group, had moved toward less drug use for virtually all of the drug use types. Given the similarities of group drug use behavior measured at baseline, this pattern alone supports the reliability of the differences created by the drug education curriculum.

**Table 4:** Drug use at six month follow-up: Comparison of means between treatment and control groups.

	Control Group *N *= 420	Drug Ed Group *N *= 389	Direction of difference	Significance Level *df = 11*	
Drug Use Variable	Mean	Mean		*F*	*p *value^*a*^

B1 Cigarettes (frequency)	1.34	1.26	Positive	3.35	**<0.001**
B2 Smokeless tobacco	1.34	1.26	Positive	3.39	**<0.001**
B3 Cigarettes (amt. smoked)	1.49	1.35	Positive	3.89	**<0.001**
B4 Alcohol	1.57	1.41	Positive	1.87	**0.040**
B5 Being drunk	1.43	1.24	Positive	1.69	**0.073**
B6 Marijuana	1.30	1.18	Positive	2.28	**0.010**
B7 Marijuana (amt. smoked)	1.18	1.13	Positive	2.12	**0.017**
B8 Sniffed glue	1.13	1.06	Positive	0.86	0.584
B9 LSD	1.05	1.04	Positive	1.12	0.339
B10 Amphetamines	1.11	1.07	Positive	2.35	**0.008**
B11 Crack	1.06	1.03	Positive	0.681	0.758
B12 Cocaine	1.08	1.03	Positive	0.97	0.471
B13 Tranquiller	1.09	1.06	Positive	0.73	0.710
B14 Barbiturates	1.10	1.05	Positive	1.07	0.380
B15 Crystal Meth	1.07	1.04	Positive	1.12	0.273
B16 Amphetamine w/o Rx	1.09	1.03	Positive	1.59	0.098
B17 Heroin	1.04	1.03	Positive	0.327	0.980
B18 Other Narcotics	1.06	1.04	Positive	1.13	0.335
B19 Ecstasy	1.05	1.03	Positive	.97	0.475
B20 Roofies	1.03	1.03	Zero	1.19	0.287
B21 GHB	1.02	1.04	Negative	2.39	**0.006**
B22 Super K	1.02	1.02	Zero	1.96	**0.030**

^*a *^Controlling for baseline differences by using an analysis of covariance with a Type III sums of squares

A number of drug use reductions achieve statistical significance. Characteristics of the specific tests indicate the effectiveness of the program. The areas of alcohol, tobacco and marijuana use in the past 30 days are particularly relevant to high school populations: Amount of cigarette use showed the strongest effect (F = 3.89, df = 11, p < 0.001,) followed by use of smokeless tobacco (F = 3.39, df = 11, p < 0.001) and cigarette frequency (F = 3.35, df = 11, p < 0.001). Frequency and amount of Marijuana were also statistically significant (F = 2.28, df = 11, p = 0.010 and F = 2.12, df = 11, p = 0.017, respectively). Differences in alcohol usage and being drunk produced marginal effects (F = 1.87, df = 11, p = 0.040 and F = 169, df = 11, p = 0.073, respectively).

Among the "hard drugs," use of amphetamines was somewhat prevalent among these youths and was significantly reduced by the curriculum (F = 2.35, df = 11, p = 0.008). Reduction in use of amphetamines without a prescription approached significance (F = 1.59, df = 11, p = 0.098).

The differences between the drug education and control groups are consistent with the literature on universal, classroom-based types of intervention [12] where drug use data is obtained by self-report and levels of substance use are high among only a small subgroup of youths [13].

### Influence of the Narconon drug education curriculum on perception of risk and attitudes about drugs or drug use compared with sites that have not yet received the curriculum

Survey questions for decisions regarding drug use, changes in perceptions of risk and attitudes regarding drug use and means of the answers for each group at follow-up along with the significance values are presented in Table 5. Corresponding percents of students answering in an anti-drug fashion are presented for each question in Tables 6, 7 and 8.

**Table 5:** Means of attitudes and beliefs responses at six month follow-up.

Attitudes and Beliefs	Control Group *N *= 421	Drug Ed Group *N *= 388	Direction of difference between treatment and control	Significance Level *df = 11*	
	Mean	Mean		*F*	*p *value^*a*^

D1 It is clear to my friends that I am committed to living a drug-free life.	2.56	2.61	More true	1.82	**0.048**
D2 I have made a final decision to stay away from marijuana.	2.65	2.66	More true	1.55	0.108
D3 I have decided that I will smoke cigarettes.	1.35	1.28	More false	1.33	**0.008**
D4 I plan to get drunk sometime in the next year.	1.72	1.54	More false	1.65	**0.003**
*How much do you think people risk harming themselves (physically or in other ways) if they...*					
D5 smoke one or more packs of cigarettes per day?^*b*^	3.61	3.60	Less risk	5.79	**<0.001**
D6 try marijuana once or twice?^*b*^	3.08	3.11	More risk	6.55	**<0.001**
D7 smoke marijuana regularly?^*b*^	3.68	3.54	More risk	9.41	**<0.001**
D8 take one or two drinks nearly every day?^*b*^	2.65	2.59	More risk	2.27	**0.010**
D9 have five or more drinks once or twice each weekend?^*b*^	1.51	1.47	More risk	1.12	0.343
*How wrong do you think it is for someone your age to...*					
D10 drink beer, wine or hard liquor (for example, vodka, whiskey or gin) regularly?	1.35	1.48	More wrong	3.15	**<0.001**
D11 smoke cigarettes?	2.45	2.45	More wrong	4.12	**<0.001**
D12 smoke marijuana?	1.54	1.30	More wrong	1.57	0.102
D13 to use LSD, cocaine, amphetamines or another illegal drug?	1.35	1.48	More wrong	3.96	**<0.001**

^*a *^Controlling for baseline differences by using an analysis of covariance with a Type III sums of squares

^*b *^The response "Can't say Drug unfamiliar" was recoded to system missing.

**Table 6:** Decisions regarding drug Use: Percent of students in each group who gave a "drug free" answer.

	Control		Drug Ed		Significance Level
	Baseline *N *= 524	6-month follow-up *N *= 418	Baseline *N *= 434	6-month follow-up *N *= 390	p value^*a*^
D1 It is clear to my friends that I am committed to living a drug-free life. (percent answering "True")	66.2%	67.5%	60.4%	69.7%	**0.048**
D2 I have made a final decision to stay away from marijuana. (percent answering "True")	75.4%	76.8%	71.9%	76.2%	0.108
D3 I have decided that I will smoke cigarettes. (percent answering "False")	77.7%	77.5%	77.0%	80.8%	**0.008**
D4 I plan to get drunk sometime in the next year. (percent answering "False")	52.7%	51.7%	55.1%	61.3%	**0.003**

^*a *^Controlling for baseline differences by using an analysis of covariance with a Type III sums of squares

**Table 7:** Perception of "harmfulness" of drugs: Percent of students in each group who answered "great risk."

	Control		Drug Ed		Significance Level
*How much do you think people risk harming themselves (physically or in other ways), if they...*	Baseline *N *= 524	6-month follow-up *N *= 418	Baseline *N *= 434	6-month follow-up *N *= 290	p value^*a*^
D5 smoke one or more packs of cigarettes per day?	66.0%	67.9%	65.2%	93.4%	**<0.001**
D6 try marijuana once or twice?	38.5%	41.6%	30.6%	53.4%	**<0.001**
D7 smoke marijuana regularly?	70.4%	72.7%	61.8%	87.2%	**<0.001**
D8 take one or two drinks nearly every day?	24.4%	33.5%	19.8%	55.2%	**0.010**
D9 have five or more drinks once or twice each weekend?	39.7%	44.7%	35.3%	71.4%	0.343

^*a *^Controlling for baseline differences by using an analysis of covariance with a Type III sums of squares

**Table 8:** Disapproval of drug use: Percent of students in each group who answered "wrong" or "very wrong."

	Control						Drug Ed						
*How wrong do you think it is for someone your age to...*	Baseline *N *= 524			6-month follow up *N *= 420			Baseline *N *= 432			6-month follow up *N *= 389			

	"wrong"	"very wrong"	total	"wrong"	"very wrong"	total	"wrong"	"very wrong"	total	"wrong"	"very wrong"	total	P value^*a*^

D10 drink beer, wine or hard liquor regularly?	22.5%	17.7%	40.3%	22.6%	27.1%	49.8%	21.5%	26.4%	47.9%	19.8%	30.6%	50.4%	**<0.001**
D11 smoke cigarettes?	28.6%	39.9%	68.5%	26.9%	41.9%	68.8%	24.3%	42.1%	66.4%	29.3%	45.0%	74.3%	**<0.001**
D12 smoke marijuana?	20.4%	60.7%	81.1%	21.2%	60.5%	81.7%	19.0%	61.3%	80.3%	17.2%	66.3%	83.5%	0.102
D13 use LSD, cocaine, amphetamines or another illegal drug?	12.8%	76.3%	89.1%	10.5%	78.8%	89.3%	9.7%	79.2%	88.9%	13.6%	72.5%	86.1%	**<0.001**

^*a *^Controlling for baseline differences by using an analysis of covariance with a Type III sums of squares

Six months after participating in the program, controlling for baseline differences, there was a much greater tendency for the control group to plan to get drunk in the year following the six-month follow-up compared with the drug education program group (F = 1.65, df = 11, p = 0.003) as well as a stronger decision to smoke cigarettes among the control group. (F = 1.33, df = 11, p = 0.008) In comparison, the drug education treatment group stated a stronger commitment to a drug free lifestyle than the control group (F = 1.82, df = 11, p = 0.048).

At six month follow-up, four out of five questions assessing risk of harm were statistically significant. Significantly more students in the drug education group indicated great risk to the question "how much do people risk harming themselves (physically or in other ways) if they try marijuana once or twice (F = 6.55, df = 11, p < 0.001) or smoke marijuana regularly (F = 9.41, df = 11, p < 0.001). These attitudes are also reflected in the developer-suggested questions with youths who received the drug education program gaining the attitude that drugs are bad (F = 1.91, df = 11, p = 0.035).

Although a greater percent of students who received the Narconon drug education curriculum indicated great risk of harm from smoking one or more packs of cigarettes per day, and having one or two drinks each day, the mean answer for that group indicated slightly less risk than answered by the control group (F = 5.79, df = 11, p < 0.001 and F = 2.27, df = 11, p = 0.010 respectively).

Among the questions assessing whether students believed drug use was "wrong" or "very wrong" for someone their age, the drug treatment group felt that dinking liquor, smoking cigarettes, and using LSD, etc., were more wrong at follow-up than did the control group (F = 3.15, df = 11, p < 0.001, for drinking liquor, F = 4.12, df = 11, p < 0.001 for smoking cigarettes, and F = 3.96, df = 11, p < 0.001 for using LSD and other drugs.)

### Competency in absorbing the material covered in the Narconon drug education curriculum compared with sites that have not yet received the curriculum

The ability of the intervention to impart knowledge was tested by examining students' ability to correctly answer nineteen items designed to assess assimilation of program content and six questions assessing their ability to apply program messages to drug use decisions and behaviors.

As shown in Table 9, six-months after receiving the drug education program, significantly more students who received the drug education curriculum were able to give answers consistent with the program content for all nineteen items, controlling for differences at baseline. Of interest, students in the drug education program improved their understanding that alcohol is a drug (F = 6.03, df = 11, p < 0.001) and that drug abuse includes both legal and illegal substances (F = 4.24, df = 11, p < 0.001). At baseline, most students had a poor appreciation of the effects of drug use on nutrient status which was corrected by the program (F = 8.79, df = 11, p < 0.001). The curriculum also corrected a common misperception about marijuana – that because it grows naturally the chemicals it contains are not harmful (F = 3.53, df = 11, p < 0.001). Students also correctly identified a major source of social influence to use drugs as media advertisements (F = 5.73, df = 11, p < 0.001). Answers to many of these questions indicate that students who received the drug education curriculum showed a greater understanding of the broad effects of drugs on the mind and body.

**Table 9:** Percent of students who gave a correct answer to program content questions.

	Control		Drug Ed		Significance Level *df = 11*	
	*N *= 524	*N *= 419	*N *= 433	*N *= 388		
	Baseline	6-month follow-up	Baseline	6-month follow-up	*F*	*p *value^*a*^

1. Drugs affect your mind only while you are taking them. (answered false)	58.8%	68.7%	58.0%	68.3%	3.21	<0.001
2. Alcohol is not a drug. (answered false)	51.3%	54.9%	53.3%	70.9%	6.03	<0.001
3. Every drug really produces just one main effect and that is what you should be concerned about. (answered false)	62.2%	69.0%	56.4%	63.9%	3.77	<0.001
4. Drug abuse only means illegal drugs. (answered false)	79.0%	80.4%	76.7%	79.1%	4.24	<0.001
5. Because marijuana grows naturally, the chemicals it contains aren't really bad for your body. (answered false)	67.9%	74.2%	60.5%	68.8%	3.53	<0.001
6. One reason youth experiment with drugs is because they are advertised in movies, television, and magazines. (answered true)	57.6%	61.6%	47.6%	64.9%	4.70	<0.001
7. All drugs change the way your body works, whether you want them to or not. (answered true)	76.1%	76.4%	65.4%	74.5%	2.15	0.015
8. Once you take a drug, it will always have the same effect each time you take it. (answered false)	48.9%	57.3%	47.3%	56.4%	3.58	<0.001
9. Drugs cause your body to use up vitamins and minerals. (answered true)	36.3%	50.4%	33.0%	72.9%	8.79	<0.001
10. Drugs can cause blank spots in your memory. (answered true)	75.2%	80.4%	66.5%	79.6%	5.06	<0.001
11. Drugs can cause a person to be sure they are doing one thing when in actual fact they are doing something else. (answered true)	68.9%	73.3%	60.0%	67.5%	5.25	<0.001
12. Hallucinogens are not as bad as other drugs. (answered false)	50.6%	57.0%	42.0%	59.0%	2.90	<0.001
13. Alcohol ads are designed only for people over 21 years of age. (answered false)	51.7%	59.2%	49.9%	58.8%	7.35	<0.001
14. Drugs can change how you feel, after a while a person on drugs can become depressed and not caring. (answered true)	76.5%	79.5%	72.3%	75.8%	3.23	<0.001
15. Once you stop drugs, it's over – they have no further effect on your body or mind. (answered false)	76.1%	77.3%	68.4%	70.9%	2.60	0.003
16. Addiction only happens once you can't say no. (answered true)	31.9%	37.9%	26.1%	24.5%	2.95	0.001
17. Its okay if you just take drugs once in a while because the body cleans all the drug stuff out in a few days. (answered false)	66.4%	69.5%	63.0%	72.9%	3.53	<0.001
18. I know how to tell if I am getting good information about drugs. (answered true)	46.9%	62.1%	49.2%	63.4%	2.56	0.003
19. A person needs to have personal goals to be happy. (answered true)	60.1%	68.3%	52.7%	69.3%	3.28	<0.001
20. It is easy for me to communicate what I think or how I feel about something. (answered true)	63.4%	70.6%	56.8%	65.2%	1.34	ns
21. I know enough about drugs to make my own decisions. (answered true)	80.0%	84.2%	76.9%	81.7%	2.77	0.002
22. I can easily resist pressures to take drugs. (answered true)	72.3%	78.8%	70.0%	74.5%	2.77	0.002
23. I have resisted pressures to take drugs before. (answered true)	66.4%	69.2%	58.9%	68.3%	.88	ns
24. In the future, I might use drugs. (answered false)	64.9%	65.9%	60.7%	60.8%	2.74	0.002
25. Drugs aren't really that bad. (answered false)	79.4%	81.6%	70.9%	75.0%	1.91	0.035

^*a *^Controlling for baseline differences by using an analysis of covariance with a Type III sums of squares

However, "addiction only happens once you can't stop," was scored "true" more often among the control group than among the treatment group (F = 2.95, df = 11, p < 0.001). This is likely due to the wording of the question itself as well as a curriculum emphasis on addiction as compulsive behavior despite known negative consequences.

Of the six questions assessing student decisions and behaviors, three produced significant change. Students in the drug prevention group were more likely to indicate that they knew enough about drugs to make decisions (F = 2.77, df = 11, p = 0.002,). Interestingly, recipients of drug prevention indicated a greater current ability to resist pressures to take drugs (F = 2.77, df = 11, p = 0.002) although the question assessing *past *resistance to drug use pressures was answered similarly between both groups at all time points. There was also a larger shift in the number of students who indicated "false" to the statement "drugs aren't really that bad" (F = 1.91, df = 11, p = 0.035).

Because a rather large percent of students in both groups answered the questions correctly at baseline, no further analysis was done to separate groups based on competency.

## Discussion

The purpose of this study was to evaluate the capacity of the Narconon drug education program to produce a long-term impact on students' drug use behaviors in a universal (all student) classroom setting. To a large degree, baseline survey responses were similar to drug use patterns seen in large national surveys. After controlling for pretest levels of use, at six months after receiving the drug prevention curriculum students in the drug education group had lower levels of current drug use than students in the comparison group. Significant reductions were observed for alcohol, tobacco, and marijuana – important categories of drug abuse for this population – as well as certain categories of "hard drugs" including controlled prescription drugs, cocaine, and ecstasy. The results in Table 4 show a clear and reliable tendency among every category tested for the drug education program to produce reductions in drug use behavior.

This is encouraging in light of the evaluation being designed to provide a "real world" test of the Narconon program under the normal conditions of operating a classroom based intervention. Inherent barriers to administering the program and evaluation while schools were in session, including assessing its effectiveness with self-report questionnaires, leads to modest measurable differences between the drug education groups and the control groups with relatively large error terms.

The use of the CSAT survey methodology does not make quantifying the reductions in drug use possible and that was not an aim of this evaluation. Importantly, by testing a universal audience, rather than selecting groups of high risk students, the mathematical differences between student responses in each category remained modest due to the majority of students indicating no drug use at baseline.

The CSAP questions testing the hypothesis that changes in attitudes and beliefs would be modified by the drug education program, argue for a mediating effect on substance use. Interestingly, the questions aimed at discerning whether new knowledge was obtained and retained over time, although indicating an overall pre-existing acquaintance with the data, nonetheless categorically produced the most statistically significant changes.

Primarily an education strategy (Center for Substance Abuse Treatment classification [14]), the Narconon program includes approaches that align with key prevention theories. Throughout the curriculum, persuasive communication is emphasized as the means to impart each component [15]. Competency enhancement is accomplished through student interaction [16] and after-school personal inspection of media and other environmental influences aimed at addressing social influences. Science based information is presented, and students complete exercises aimed at developing their ability to assess the correctness of messages presented as information from a variety of sources.

Originally researched on cigarette use by Evans and colleagues in 1976, social influence theory was one of the first strategies to produce an impact on drug use behavior. This theory posits that alcohol and other drug use among young people is primarily a social behavior strongly influenced by social motives, a complex and reciprocal interaction between both personal and environmental factors including both overt and covert pressure from friends and others to conform to what is depicted as the group norm. A major departure from previous approaches to tobacco, alcohol, and other drug abuse prevention; Evans work emphasized increasing awareness of the various social pressures promoting drug use, including media influences [17,18].

One well-popularized aspect of today's social influences model is the focus on social resistance skills training. However, programs based primarily on resistance training have shown mixed results [19,20]. While this is not a focus of the Narconon program, students who received the curriculum were more likely to say they could now resist pressures to use drugs compared with those who did not receive this program. Interestingly, both groups answered similarly about their ability to resist pressures in the past.

Instead of directly practicing resistance skills, the Narconon drug education curriculum provides an opportunity for youth to inspect a myriad of positive, negative and often conflicting messages regarding drugs and their abuse, messages that often include incorrect and conflicting information about drugs and their effects. Program developers believe that prevention effectiveness is currently compromised by the pervasiveness of conflicting messages, including popular prevention approaches that do not communicate a consistent message.

Attempts to promote abstinence contrast with other messages heard in and out of school. For example, the notion that "everyone will experiment" has lead to various, sometimes controversial, practices aimed at reducing harm [21]. Goodstadt argues that dichotomies such as "licit" versus "illicit" drugs, or simply "good" versus "bad" drugs, result in ambiguities and problems [22]. Petosa adds that legal definitions designating certain recreational drug as "licit" for adults but "illicit" for adolescents may encourage young people to use those drugs to demonstrate their transition to adulthood [23]. The current prevalence of media advertising for prescription medications sends another powerful message [24], one complicated by the fact that commonly prescribed medications are too often used in ways substantially inconsistent with diagnostic guidelines [25,26].

Although students may "know" a certain datum about drugs, conflicting messages such as these may cause that datum to be minimized or rejected entirely unless placed in correct context or inspected relative to other information. To address this, the program teaches about the often subtle pro-drug advertising and other environmental messages aimed at increasing tobacco, alcohol and other drug consumption; contrasting these pro-drug messages with true scientific facts about drug effects on the body, mind, emotions, and enjoyment.

Program facilitators purposefully encourage students to arrive at their own conclusions regarding the data presented based on each student's own observation of the topic under discussion. Facilitators do not tell students what to think, rather, they teach students how to observe.

Another environmental influence addressed by the Narconon program includes more accurate awareness of family and peer drug use patterns. The program includes modules to review and discuss personal observations and provide opportunity for youth to work out what are correct and pro-survival norms.

Media, family, peer and other environmental influences become the subject of competency enhancement activities included in the Narconon curriculum. Competency to observe is applied during after-school practicals and becomes subject of the subsequent group discussion. These take home assignments and classroom activities are also aimed at developing broader personal and social skills with peers, family and community members. Research supports the use of activities that improve interpersonal relations, self esteem, communication, and other skills as directly applicable to substance use as well as many other adolescent problems. Such activities appear to generally enhance program effects [27,28].

With respect to the importance of knowledge, while many early prevention programs gave individuals accurate facts about the harmful effects of alcohol and other drugs, theorizing that those individuals would reduce or avoid drug use because it was in their own best interest to do so, studies of this generic information-only or awareness model have led to one of the very few universally agreed-upon facts in the prevention field: That is, for the vast majority of individuals, simple awareness through passive receipt of health information is not enough to lead them to alter their present behavior or reduce their present or future use of drugs [29,30].

According to Botvin and Botvin [12,16]., inclusion of information remains a necessary component of substance abuse education, although information alone is not sufficient to reduce or prevent use. Evans stresses the importance of attention and comprehension of the contents of the message [15]. Narconon program developers posit that true information correctly communicated can lead to changed behavior by changing the perceived value or social acceptance of that information.

Since inception, Narconon prevention training materials have emphasized *correct communication *of information and interaction with the communicator. Facilitator training aligns with the five component *communication persuasion model *described by McGuire [31]. According to this theory, to be effective an educator must get and hold the listeners' attention, must be understandable (comprehension), must elicit acceptance on the part of the person exposed to the message (yielding), the acceptance must be retained over time (retention), and thereby be translated into action in appropriate situations. Testing the ability to choose a correct answer only begins to answer the question of the perceived value and usefulness of that information. To that end, the incorporation of persuasive communication into facilitator training and multi-media program components is suggestive. In theory, the communication of science-based information regarding the nature and effects of drugs can assist students in developing judgment and awareness, but only to the extent that the message sent is very real to youths and delivered in a way that students respect and can appreciate. Measurements of student satisfaction that include affective reactions (e.g. enjoyment, content value) should be further explored as they may reveal important shifts in perceptions about the information itself that would not be detected in simple "true/false" questions.

This theory is supported by a previous evaluation of 1045 post-program student surveys, published in 1995, with findings that the Narconon program format was engaging and appreciated by youths [32]. Participants also reported heightened perceptions of risk – including a shift in attitude among the borderline group of students who held the view that they might use drugs in the future. Eighty six percent of the students in this category stated that the session they had attended changed their mind; most stating that they were now more concerned about the effects of drugs or that they had not realized that drugs were so damaging.

In addition to analyzing elements of content and implementation, a recent synthesis of characteristics common to exemplary prevention programs by Winters, *et al. *[33] raises the issue of management structure and sustainability. Narconon International's corporate and regional offices provide centralized management and assistance to ensure that local prevention offices receive meaningful attention and support. In addition to the questionnaire used in this study, Narconon program staff continued to collect their own feedback evaluations for ongoing quality management. Staff interaction with teachers and community members helped the schools further reinforce the prevention messages.

The report by Winters, *et al. *[33] points out the broad lack of programs aimed at high school years and, interestingly, the need for multiple sessions in future years to reinforce the message. The Narconon high school curriculum helps fill this need. Existing materials for younger ages should also be developed into an age appropriate curriculum to provide a continuum of educational resources. As the program further develops its training materials for professional facilitators it may consider also making them appropriate for peer leader groups who may particularly benefit through improved communication skills. The program should also develop appropriate universal booster sessions and provide educator consultation.

Project findings may have policy implications regarding both setting goals and objectives for prevention programs as well as evaluating their success. For example, the Safe and Drug-Free Schools and Communities act of 1994 includes "slow recently increasing rates of alcohol and drug use among school-aged children by 2000" among the six performance indicators chosen for assessing program accomplishments. It also expects prevention to "realize continuous improvement in the percentage of students reporting negative attitudes toward drug and alcohol use between now and 2002". Further, this act is subject to requirements of the Government Performance and Results Act of 1993 (GPRA) in requiring local and state education agencies to monitor program effectiveness, for which the CSAT instrument is a recommended tool sanctioned by the National Institute on Drug Abuse (NIDA), and the Substance Abuse and Mental health Services Administration (SAMHSA). Unfortunately, the instrument is unable to quantify change in drug use and does not assess completely the factors that might lead to such a change, factors that may include change in knowledge and the perceived value of that knowledge.

As current youth drug use levels remain high, it is clear that much more remains to be learned regarding effective drug abuse prevention. What works best; what goals additional to reduction in youth drug use – if achieved – constitute an effective program; how to measure achievement and the extent to which a school-based implementation strategy can counter other influences remains under discussion.

## Conclusion

As an intensive, eight-module, educational curriculum, the Narconon program has thorough grounding in theory and substance abuse etiology, incorporating several important and historically successful prevention components. This supports the prediction that participants in this classroom-based program would change their behavior regarding drugs of abuse. Further, the Narconon network provides a strong organizational structure to foster sustainable and high fidelity program implementation.

In this evaluation, the Narconon drug education curriculum produced reliable reductions in drug use a full six months after completion of the drug education program and in every category of drug use tested. A third of these questions – those assessing the drugs most commonly used by youths; alcohol, tobacco and marijuana as well as "hard drugs" – showed statistically significant reductions in use. The reductions achieved with both amphetamines and non-prescription use of amphetamines are important given recent increases in availability and initiation of these drugs. The reliability of the reductions measured in drug abuse behavior provide the most relevant support for the Narconon drug education curriculum.

The program's ability to produce reductions in drug use behavior appears to be through correcting prevalent but false messages while empowering youth to observe, draw their own conclusions, and potentially also improves interpersonal skills contributing to the development of appropriate group norms. These changes may result in shifts in perception of risk and corrected attitudes as individuals and as a group. However, the mechanisms of action for this program should be further explored using sensitive instruments and analyses designed to test this hypothesis. Although the CSAP questionnaire underwent an extensive development process, isolating effective components of drug prevention programs may require a more robust methodology, particularly in light of the theory constructs of this program.

The Narconon drug education curriculum for high school grades shows clearly positive results and sends an important and powerful message promoting abstinence. Given the significant reductions in drug use behavior, the scientific content and social influence theory underlying the program materials and their implementation, and the strong, centralized management by Narconon International, this program is very promising and fills a vital need in substance abuse prevention.

## Competing interests

M Cecchini wishes to disclose that between 2000–2002 she was the Executive Director of a Narconon center engaged in delivering substance abuse prevention programs; familiarity with program operations made it possible to coordinate independent field data collection with ongoing prevention efforts and assisted in describing the history and development of the program.

## Authors' contributions

RL is Principal Investigator and developed the study design, statistical analysis and interpretation, and drafted sections of the manuscript.

MC coordinated the independent field data collection staff with scheduled drug education program delivery, ensured compliance with procedures to protect human subjects, and drafted sections of the manuscript.

Both authors read and approved the final manuscript.

Please send all reprint/proof correspondence to Marie Cecchini, 10841 Wescott Avenue, Sunland CA 91040.
